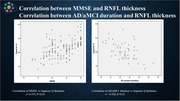# Analysis of Macular thickness and retinal nerve fiber layer by using of spectrum domain‐optical coherence tomography in patients with Alzheimer’s disease and amnestic mild cognitive impairment

**DOI:** 10.1002/alz.085677

**Published:** 2025-01-09

**Authors:** Bon D Ku, Kyung‐Hoon Shin, Do‐Gyun Kim

**Affiliations:** ^1^ Institute for Cognitive Intervention, Incheon Korea, Republic of (South); ^2^ International St. Mary’s Hospital, Catholic Kwandong University College of Medicine, Incheon Korea, Republic of (South); ^3^ Ophthalmology, Kim’s Eye’s Hospital, Konyang University, South Korea, Seoul Korea, Republic of (South); ^4^ Ophthalmology, Myongji Hospital, Hanyang University College of Medicine, Goyang Korea, Republic of (South)

## Abstract

**Background:**

Spectrum Domain‐Optical Coherence Tomography (SD‐OCT) is a non‐invasive technology that acquires cross‐sectional images of retinal structures allowing neural fundus integrity assessment. Macular thickness and retinal nerve fiber layer (RNFL) thickness measured by an SD‐OCT have been used as a indicator of Alzheimer's disease (AD) and amnestic mild cognitive impairment (aMCI). However which portion of retinal RNFL is the most sensitive area among normal control, aMCI and AD is not clear yet. The purpose of this study is to demonstrate that RNFL thickness is a useful indicator and which portion of reninal RNFL is the most sensitive area among normal control.

**Method:**

In a cross‐sectional study we consecutively recruited 53 patients with AD, 58 with aMCI, and 54 normal controls. AD‐OCT was performed in all of them to measure circumpapillary macular thickness in the 9 sectors (fovea, temporal outer superior outer, nasal outer, inferior outer, temporal inner, superior inner, nasal inner, inferior inner). We made 4 RNFL quadrant area as following: superior (superior outer+superior inner), inferior (inferior outer+ inferior inner), nasal (nasal outer + nasal inner), temporal (temporal outer + temporal inner). We also evaluated the correlation of the RNFL thickness and MMSE score and disease duration of the patients

**Result:**

Average macular thickness and 9 sectors of RFNL thickness were not significant among the group in our patients. In quadrant analysis, however superior quadrant RNFL thickness showed significant differences among groups (109.98±12.01 um, 106.83±10.05 um 101.25±11.90 um in normal control, aMCI and AD respectively, p<0.01). The RNFL thinning of the superior quadrant showed a significant correlation with MMSE score (r=0.555, p<0.01) and AD duration (r=‐0.528, p<0.01).

**Conclusion:**

The superior quadrant RNFL thickness is the most sensitive among the group and as well as we know this is not reported before. This finding could suggest superior quadrant retinal RNFL by SD‐OCT could be a useful marker of AD and a MCI for early detection and monitoring of disease progression. Also distinct correlation of RNFL thinning in superior quadrant and MMSE score and disease duration can imply that the severity of dementia can be inferred from RNFL thickness in superior quadrant.